# Anti-smoking social norms are associated with increased cessation behaviours among lower and higher socioeconomic status smokers: A population-based cohort study

**DOI:** 10.1371/journal.pone.0208950

**Published:** 2018-12-12

**Authors:** Danielle A. J. M. Schoenaker, Emily Brennan, Melanie A. Wakefield, Sarah J. Durkin

**Affiliations:** Centre for Behavioural Research in Cancer, Cancer Council Victoria, Melbourne, Australia; Universita degli Studi di Napoli Federico II, ITALY

## Abstract

**Background:**

Social denormalisation of smoking can provide an environment that helps smokers to quit. This study examined which of three measures of anti-smoking social norms have the greatest influence on quitting-related cognitions and behaviours, and if this influence differs according to socioeconomic status (SES).

**Methods:**

The Victorian Tracking Survey measured social norms among 1,348 (n(weighted) = 1,373) Australian adult smokers (aged 18–59) between 2012 and 2014, who were followed-up one week later. Weighted logistic regression analyses examined prospective associations of baseline subjective (family and friends’ disapproval of smoking), injunctive (feeling embarrassed about being a smoker) and descriptive norms (living with someone who tried to quit in the past 12 months), with quitting-related cognitions and behaviours at follow-up. Data were weighted to account for telephony status (landline or mobile phone), sex and age. Analyses were adjusted for demographic characteristics, addiction level, tobacco control policies and quitting-related outcomes measured at baseline. Differences in associations between lower and higher SES smokers (based on educational attainment and area-based disadvantage) were examined through interaction terms and stratified analyses.

**Results:**

Sixty-four percent of participants (n(weighted) = 872) perceived disapproval from family and friends, 31% (n(weighted) = 419) felt embarrassed to be a smoker, and 11% (n(weighted) = 155) lived with a recent quitter. All three norms were associated with having set a firm date to quit in the next month and with engaging in smoking limiting behaviours. Embarrassment was also associated with an increased likelihood of talking about quitting and with making a quit attempt. Associations were mostly comparable for lower and higher SES smokers, with no significant negative rebound effects overall or among subgroups.

**Conclusions:**

These findings indicate close others’ disapproval and feelings of embarrassment most strongly motivate smokers to try to quit. If tobacco control policies or media campaigns further denormalise smoking, there should be no reason for concern that such denormalisation undermines cessation behaviours.

## Introduction

Social norms are acknowledged as playing a key role in population behaviour change [1, 2]. The shifts in social norms and social pressure that occur as a result of major tobacco control interventions and campaigns can strengthen smokers’ motivation to quit and commitment to staying quit [1, 3–5]. Over the 2012 to 2014 period examined in this study, several key tobacco control policy changes occurred in Australia, including the introduction of plain packaging of tobacco products with larger and refreshed pictorial health warnings, the implementation of two 12.5% annual tax increases, and variable levels of mass media campaign activity to warn of the serious harms of smoking [6–8]. This study aimed to examine the influence of social norms on quitting thoughts and behaviours over this period, independent of the direct influence of these policy interventions and campaigns.

Several cohort studies of adult smokers have found that higher levels of anti-smoking social norms and denormalisation of smoking were prospectively associated with an increased likelihood of intending to quit and making a quit attempt [3, 4, 9–11]. While most studies considered multiple measures of tobacco denormalisation and combined various social norms into one scale [4, 9, 11], less attention has been given to the differential impact of various types of social norms. This hinders comparison of findings across populations and the identification of specific aspects of social norms that most strongly predict quitting behaviours.

One cross-sectional study that did examine the influence of subjective (what the smoker perceives others want) and injunctive norms (whether the smoker perceives smoking is an acceptable behaviour) separately from descriptive norms (what the smoker perceives most people do and/or exposure to people in their close environment who smoke and/or quit), found that subjective and injunctive norms were more strongly associated with intentions to quit than descriptive norms [12]. Moreover, two studies have examined individual subjective and injunctive norms separately, showing that perceived disapproval from significant others was more strongly related to quit intentions and attempts compared with perceived societal disapproval [3, 13]. Positive and negative reactions from close family or friends influence people’s emotions [2, 14], and having the perception that close family or friends disapprove of someone’s smoking may provide a strong incentive to try to change that behaviour. There is some indication that this subjective norm may be particularly important. Cross-sectional studies have found subjective norms (important others’ disapproval of smoking) to be more strongly associated with quitting intentions and behaviours than injunctive norms (perceptions of what broader society thinks is acceptable) [12, 13]. This is further supported by a longitudinal study by Rennen and colleagues who found that disapproval from people important to the smoker, but not societal disapproval, was associated with making a quit attempt during one year of follow-up [3].

Although studies have included a variety of measures tapping different types of social norms [3, 4, 9, 10, 13], only a few have examined internalisation of social norms [10]. While subjective, injunctive and descriptive norm perceptions can all be considered “external”, resulting from exposure to disapproving others, to smoking restrictions, and/or to people in their close environment who smoke and/or quit [2], internalised social norms about smoking result in “self-conscious” feelings of guilt and embarrassment generated by self-reflection and self-evaluation that one has fallen short of what one feels one ought to do [2, 15]. A limited number of studies suggest that experiencing guilt and embarrassment about smoking can be motivational. For example, a few qualitative, cross-sectional quantitative and pre-post experimental studies have found that self-blame and guilt about smoking behaviour were associated with stronger self-efficacy to quit [16], were protective of relapse [17], and increased quitting intentions [18]. In addition, a recent cross-sectional telephone survey of over 8,000 adult smokers and recent quitters from the Australian state of Victoria demonstrated that prior exposure to anti-tobacco advertisements evoking multiple negative emotions (fear, sadness and/or guilt) increased the likelihood of making a quit attempt [19]. A natural history study examining daily-reported quitting cues over a 12-week period found the most common quitting cue was feeling embarrassed about smoking, with almost half reporting this each week [10]. The next most common cues were the cost of smoking, media messages and someone asking them to quit or mentioning smoking harms. This study found that the cumulative number of cues over the past 7-days prospectively predicted quit attempts [10]. Thus, internalised social norms that evoke feelings of guilt and embarrassment about smoking may be potent motivators of quit attempts, however, evidence on the effects of internalisation of social norms is currently mixed, and based only on qualitative and cross-sectional quantitative studies. Further research is therefore needed using prospective cohort studies.

It is also important to consider the potential negative consequences of public denormalisation of smoking [20, 21]. While both externally and internally generated negative emotions may prompt adherence to the accepted norms of the immediate social environment to re-gain approval from others and avoid these negative feelings [2, 15], it has been hypothesised that when these feelings become overwhelming they may have undesirable impacts on behaviour [20, 21]. Evidence for this potential negative effect comes from qualitative and cross-sectional quantitative studies indicating that smoke-free regulations and perceived disapproval from others may lead to feelings of punishment, victimisation and demoralisation [22], reduced participation in social activities [23], and nondisclosure of smoking status to doctors or other healthcare providers [24]. However, there is no evidence from cohort studies on whether smokers who experience negative emotions subsequently have lower quit motivation or attempt rates. This study therefore aimed to explore the influence of anti-smoking subjective, injunctive and descriptive norms on a range of subsequent quitting-related cognitions and behaviours.

### Socio-economic differences

Findings from qualitative studies have suggested that lower socioeconomic status (SES) smokers are more likely to accept that their smoking presents a risk to others and are more likely to experience subtle and overt social disapproval of smoking when they move out of their own social context into a wider non-smoking one. However, they are less likely to experience this disapproval within their close social networks where smoking is more normalised, compared with higher SES smokers [25–27]. In an illustrative study of over 2,500 smokers, Sorenson and colleagues found that compared with other workers, blue-collar workers reported less pressure to quit, lower social support for quitting, and greater acceptability of smoking among their co-workers [11]. Higher SES smokers on the other hand have reported being more likely to comply with anti-smoking social norms by concealing their behaviour from family, colleagues and friends [25, 27, 28]. Other qualitative studies have indicated that smokers with higher education and income were more likely to perceive disapproval, guilt and embarrassment compared with those of lower SES [9, 28]. This suggests that stronger internalisation of these norms against smoking among higher SES groups may result in disparities in smoking [29] by motivating greater cessation activity in these higher SES groups. However, most of the current evidence about whether lower or higher SES smokers are more likely to perceive social pressure is based on small qualitative studies, and so further research is needed to explore the prevalence of subjective, internalised injunctive and close descriptive norms among lower and higher SES smokers, and associations with quitting-related attitudes and behaviours separately in these subgroups. This examination will help determine if policies that enhance smoking denormalisation promote cessation among all smokers.

The aims of the present study were therefore to describe the prevalence of adult smokers’ smoking-related perceptions of disapproval from family and friends, feelings of embarrassment and close others’ quitting activity overall and across SES subgroups, to examine associations of these social norms with quitting self-efficacy, urgency, intentions and behaviours, and to determine if these associations differed by SES.

## Materials and methods

### Study design and participants

The Victorian Tracking Survey (VTS) is a continuous cross-sectional telephone survey of Victorian adults aged 18–59 years who were smokers (currently smoked daily or weekly, or smoked monthly or less-than-monthly but self-identified as a smoker) or recent quitters (quit in the last year). The VTS monitors recall of state-funded anti-tobacco mass media campaigns and smoking-related cognitions and behaviours. Participants were recruited for the baseline interview using a dual-frame probability sampling design, with half of all participants approached via landline and half via mobile phone random digit dialling. Telephone interviews were conducted in English, and only participants who reported watching any free-to-air commercial television on an average weekday were eligible to participate. An average of 174 interviews were completed each month from January 2012 to November 2015. Data collection was suspended for the late December to early January summer holiday period. The mean monthly baseline response rate, adjusted for those who declined to be formally screened but may have been in-scope for the interview, was 42%.

For the first three years of this survey (January 2012 to November 2014) participants were contacted for participation in a follow-up interview approximately one week after the baseline interview (median 8 days, range 7 to 23 days), only if during their baseline interview they recalled one of the state-funded advertisements that had been broadcast. Of 2,363 baseline smokers who were eligible for follow-up (i.e. recalled an advertisement and were not a recent quitter), 1,434 were successfully re-interviewed (61%) (n(weighted) = 1,459). A total of 1,348 participants had complete data on all predictors, outcomes and covariates and were included in this study (n(weighted) = 1,373).

At baseline, the VTS measured anti-smoking social norms (predictor variables), and demographic characteristics, addiction level, tobacco control policies and quitting-related cognitions and behaviours (covariates). At approximately one week follow-up, quitting-related cognitions and behaviours were assessed for the second time (outcome variables).

### Quitting-related outcome measures at follow-up

At follow-up, to measure self-efficacy to quit participants were asked to indicate on a 10-point scale how confident they were that they could quit smoking for good in the next three months if they wanted to (1 ‘not at all confident’ to 10 ‘extremely confident’). To measure urgency of quitting they were asked how they would rate quitting as a priority in their life (1 ‘lowest priority’ to 10 ‘highest priority’). Ratings of nine or 10 were defined as being highly confident to quit in the next three months and having quitting as a high priority. Participants’ intentions to quit were measured by asking participants if they had set a firm date to quit in the next month [30].

Smoking behaviours between baseline and follow-up were assessed, including three behaviours to limit smoking that were combined to indicate if participants engaged in at least one (vs. none) of these smoking limiting behaviours. Participants were asked whether, since baseline, they had tried to limit the number of cigarettes; had stubbed out a cigarette before finishing it as a result of having thoughts about the harms of smoking; or had not had a cigarette despite having the urge to smoke. The Cronbach’s alpha for these three items was 0.85, which is a measure of internal consistency and indicates these smoking limiting behaviours are closely related. Participants also indicated if, since baseline, they had discussed quitting with family or friends, and if they had sought help to quit (at least one of: called the Quitline, consulted a doctor, used nicotine replacement therapy or other quit smoking medication, and/or researched quitting on the internet). Quit attempts were assessed by asking participants to indicate how many times, if any, they tried to quit smoking for at least 24 hours since baseline, which was dichotomised as attempted to quit, or not. These quit attempts could be successful or unsuccessful. Because of the low proportion of participants who had quit and remained abstinent until the follow-up interview (on average, 8 days later; 2.7%), we were not able to examine sustained quitting as a separate outcome.

### Anti-smoking social norms

At the baseline interview, participants were asked to rate the statements “My closest friends and family members disapprove of my smoking” and “I feel embarrassed to tell people I’m a smoker” on a five-point scale ranging from ‘strongly agree’ (1) to ‘strongly disagree’ (5). Positive responses to ‘strongly agree’ or ‘agree’ were coded “1” and used to define disapproval and embarrassment, with all other responses coded as “0”. Participants were also asked “Counting yourself, how many people in your household quit smoking in the past 12 months?”. After subtracting participants who tried to quit themselves in the past 12 months, responses were categorised as those living with at least one recent quitter, or living in a household with no recent quitting activity.

### Socioeconomic subgroups

The VTS collected information on individual-level educational attainment, while no information was available on individual-level occupation or income. SES subgroups were therefore based on individual-level education and on the area-level Socio-Economic Index for Areas (SEIFA)-Disadvantage index. Education was categorised as low education, defined as those who had completed year 12 education or less (secondary school), or high education, including those who had completed some higher education. The SEIFA-index was developed by the Australian Bureau of Statistics based on 2011 Census data of residential areas [31] and ranks geographical areas (postcodes) on a scale from high disadvantage to low disadvantage based on income, education, occupation and housing conditions in the area. Participants living in the lowest 40% of residential areas in Victoria were categorised as high disadvantage, and those living in the top 60% of areas as low disadvantage. For analysis, low SES was defined as those with low education and who lived in a high disadvantage area, mid SES as those with either low education who lived in a low disadvantage area or those with high education who lived in a high disadvantage area, and high SES as those with high education and who lived in a low disadvantage area.

### Covariates

Main analyses were adjusted for baseline covariates including sex, age and SES. Addiction level was based on the Heaviness of Smoking Index [32]. This index was created based on responses to questions “On the days that you smoke, how soon after you wake up do you have your first cigarette?” (coded as ‘0’ for after 60 minutes; ‘1’ for 31–60 minutes; ‘2’ for 6–30 minutes; and ‘3’ for within five minutes) and “How many cigarettes do you typically smoke per day?” (coded as ‘0’ for ≤10; ‘1’ for 11–20; ‘2’ for 21–30 and ‘3’ for ≥31 cigarettes per day). Low addiction was defined as 0–2 points, moderate addiction as 3–4 points and high addiction as 5–6 points [32].

Covariates also included change in cigarette costliness (the average price of the 10 market-leading cigarette brands divided by average weekly earnings) in line with methods used in recent studies [19, 33, 34]. Changes in cigarette costliness reflect the three-month period following increases in price related to either indexation of, or real increases in, excise/customs duty (i.e. the month of change plus the following two months), based on the assumption that costliness changes exert most influence on quitting in the three months that follow a price increase [35]. To account for the influence of the implementation of plain packaging, we included a binary variable indicating if participants were interviewed before the implementation or during the transition months (‘0’ for January 2012 –November 2012), or post-implementation (‘1’ for December 2012 –December 2014) [36].

Participants’ behaviours may also have been influenced by anti-tobacco television advertisement exposure. Data on anti-tobacco advertisements appearing on television between January 2012 and November 2014 were obtained from Nielsen/OzTAM Pty Ltd (North Sydney, Australia). The measure of advertisement exposure was based on Gross Rating Points (GRPs), which reflects the average potential exposure and is calculated as the product of the percentage of the audience exposed to an advertisement (reach) and the average number of times the audience is exposed (frequency). We used advertisement exposure during the month of the interview, and the two preceding months, based on previous research that has indicated that advertisement effects on quitting-related behaviours occur up to two to three months after exposure [37].

To account for the influence of pre-existing sample differences in baseline levels of quitting-related outcomes, models were adjusted for the baseline equivalent of each outcome measure if this was available. Questions about confidence to quit, quitting priority, setting a firm date to quit, seeking help to quit and discussing quitting with family or friends were identical at baseline and follow-up, however the baseline interview did not include questions on smoking limiting behaviours. Instead participants were asked to report whether any of these smoking limiting behaviours occurred between baseline and follow-up. All models (including for the quit attempts outcome) were adjusted for time since previous quit attempt at baseline. To account for differences in the timing of the follow-up interviews we included a variable on the number of days between the baseline and follow-up interview.

A time variable (month and year of baseline interview) was tested but not included in the final analysis, as this was collinear with plain packaging implementation (variance inflation factor (VIF) >5 in all models). However, in sensitivity analyses including the time variable, the pattern of findings was comparable to that from the final model that excluded the time variable.

### Statistical analyses

Data were weighted to account for telephony status (landline or mobile phone), sex and age, according to estimates of these distributions from a representative sample of smokers and recent quitters collected in the Victorian Smoking and Health Survey in November/early December each year [38]. All analyses were conducted using StataV14.1 [39] using weighted data (with the svy command and ‘p’ weights).

Baseline demographic and smoking-related characteristics were described for all participants included in the current study, and characteristics of low, mid and high SES participants were described and compared using chi-square tests.

Logistic regression analyses examined associations of close family and friends’ disapproval, embarrassment about being a smoker and living with a recent quitter, with quitting-related outcomes. Each social norm was examined in a separate model, adjusted for covariates.

Consistency of the social norms and quitting-related behaviour associations across SES subgroups was also examined using logistic regression. Interaction terms were included in the respective models for disapproval, embarrassment and household quitting activity with the SES measure, and a p-value of <0.10 for the post-model Wald test for the interaction was considered to indicate a potentially relevant interaction. Stratified logistic regression analyses were performed by SES subgroups for all outcomes to provide separate odds ratios to enable visualisation of potential differences between subgroups.

### Ethics approval

The study was approved by the Human Research Ethics Committee of Cancer Council Victoria (HREC 1104). The data were analysed anonymously.

## Results

### Participant characteristics

At baseline, just under half the final sample of smokers were female (n(weighted) = 593, 43%) and almost three-quarters were aged 30–59 years (n(weighted) = 975, 71%) (**Table 1**). Higher education was completed by 49% of participants (n(weighted) = 671), and 61% lived in a higher SES area (n(weighted) = 840). The majority of participants had low or moderate addiction levels (n(weighted) = 1,275, 93%), and had previously made a quit attempt (n(weighted) = 1,120, 82%). About a third of participants were interviewed during the ten months before implementation or during the transition to plain packaging (n(weighted) = 402, 29%). Almost one third of participants were interviewed in a three-month period after cigarettes became more costly (n(weighted) = 413, 30%).

Compared with participants who were eligible to participate in the follow-up interview but were not included in this study, participants who were included were more likely to be older (n(weighted) = 446, 35% vs. n(weighted) = 294, 29% aged 45–59 years) and less likely to be under 30 years of age (n(weighted) = 334, 26% vs. n(weighted) = 370, 37%), and more likely to have moderate addiction levels (n(weighted) = 437, 34% vs. n(weighted) = 271, 27%). The prevalence of anti-smoking social norms was not significantly different among smokers who were included in this study compared to those who were lost at follow-up (n(weighted) = 809, 63% vs. n(weighted) = 608, 61% experienced disapproval [p = 0.33], n(weighted) = 405, 32% vs. n(weighted) = 292, 29% felt embarrassed [p = 0.19] and n(weighted) = 137, 11% and n(weighted) = 136, 14% lived with a recent quitter [p = 0.08], respectively).

**Table 1:** Baseline characteristics and quitting-related cognitions and behaviours of study participants.

	Total n(weighted) = 1,373	Low SES n(weighted) = 314	Mid SES n(weighted) = 607	High SES n(weighted) = 452	
	n(weighted) (%)	n(weighted) (%)	n(weighted) (%)	n(weighted) (%)	p-value^a^
*Baseline covariates*					
Female	593 (43.2)	138 (43.9)	253 (41.7)	201 (44.6)	0.72
Age					0.005
18–29 years	399 (29.0)	81 (25.8)	191 (31.5)	127 (28.0)	
30–44 years	533 (38.8)	109 (34.6)	215 (35.4)	209 (46.2)	
45–59 years	442 (32.2)	125 (39.6)	201 (33.1)	117 (25.9)	
Plain packaging					0.38
Pre-implementation	402 (29.3)	80 (25.5)	182 (30.1)	140 (30.9)	
Post-implementation	972 (70.7)	235 (74.5)	424 (70.0)	313 (69.2)	
Change in cigarette costliness					0.55
No change in costliness	961 (69.9)	228 (72.4)	426 (70.2)	307 (67.9)	
Increase in costliness	413 (30.1)	87 (27.7)	181 (29.8)	145 (32.1)	
Addiction level					<0.0001
Low addiction	818 (59.6)	145 (46.1)	360 (59.3)	313 (69.3)	
Moderate addiction	457 (33.3)	135 (42.9)	202 (33.3)	120 (26.6)	
High addiction	98 (7.1)	35 (11.0)	45 (7.4)	19 (4.1)	
Time since previous quit attempt					0.48
No previous attempt	253 (18.4)	61 (19.3)	109 (18.0)	83 (18.4)	
Up to 3 months ago	229 (16.7)	46 (14.8)	104 (17.1)	79 (17.4)	
3–12 months ago	273 (19.9)	55 (17.5)	112 (18.5)	106 (23.5)	
More than 12 months ago	618 (45.0)	153 (48.5)	281 (46.4)	184 (40.6)	
*Follow-up outcomes*					
Quitting is a high priority	412 (30.0)	86 (27.4)	177 (29.1)	149 (33.0)	0.31
Highly confident to quit in the next 3 months	296 (21.5)	59 (18.9)	141 (23.2)	96 (21.2)	0.47
Set a firm date to quit in the next month	78 (5.7)	6 (1.9)	42 (7.0)	30 (6.7)	0.008
Since baseline, engaged in smoking limiting behaviours	979 (71.3)	215 (68.2)	420 (69.2)	344 (76.2)	0.07
Since baseline, discussed quitting with family or friends	384 (27.9)	83 (26.4)	168 (27.7)	132 (29.3)	0.76
Since baseline, sought help to quit and/or used NRT or quit smoking medication	204 (14.9)	36 (11.3)	89 (14.7)	79 (17.6)	0.13
Since baseline, attempted to quit	81 (5.9)	8 (2.4)	40 (6.6)	33 (7.4)	0.05

SEIFA, Socio-Economic Index For Areas; SES, socioeconomic status

^a^ p-values from chi-square tests comparing distributions across low SES, mid SES and high SES subgroups

### Anti-smoking social norms and quitting-related behaviours

Almost two thirds of participants (n(weighted) = 872, 64%) agreed with the statement that their closest friends and family members disapproved of their smoking. Feeling embarrassed to tell people they are a smoker and living in a household with a recent quitter were less common at 31% (n(weighted) = 419) and 11% (n(weighted) = 155), respectively. All three social norms were slightly less common among low compared with mid and high SES smokers, but these differences were not statistically significant (p >0.05). The three social norms each reflect a different aspect of smokers’ experiences, based on the findings that less than a quarter of participants (n(weighted) = 333, 24%) experienced both disapproval and feeling embarrassed, and 7% (n(weighted) = 92) and 5% (n(weighted) = 62) reported both disapproval and household quitting activity and embarrassment and household quitting activity, respectively.

Baseline disapproval and embarrassment each approximately doubled the likelihood of having quitting as a priority, and baseline disapproval, embarrassment and household quitting activity each more than doubled the likelihood of having set a firm date to quit in the next month (**Table 2**). In addition, baseline disapproval and household quitting activity, and to a greater extent embarrassment, increased the likelihood of engaging in smoking limiting behaviours (**Table 3**). Embarrassment, but not disapproval or living with a recent quitter, also increased the likelihood of discussing quitting with family or friends and making a quit attempt since baseline (Table 3). Social norms were not associated with high confidence to quit in the next 3 months (Table 2) or seeking help to quit (Table 3).

**Table 2:** Odds ratios and 95% confidence intervals from logistic regression analyses examining associations between baseline social norms, and quitting-related cognitions and intentions at follow-up.

	Quitting is a high priority N (weighted) = 1,373	Highly confident to quit in the next 3 months N (weighted) = 1, 373	Set a firm date to quit in the next month N (weighted) = 1, 373
	Odds ratio (95% CI)	Odds ratio (95% CI)	Odds ratio (95% CI)
Friends and family disapprove of my smoking	2.05 (1.41, 2.98)*	1.11 (0.74, 1.66)	2.19 (1.01, 4.74)*
Feel embarrassed to tell people I’m a smoker	2.02 (1.41, 2.89)*	1.39 (0.87, 2.20)	2.38 (1.30, 4.35)*
Quitting activity in the household	1.63 (0.97, 2.74)	1.04 (0.57, 1.92)	3.32 (1.47, 7.51)*

Each social norm was examined in a separate model. All models were adjusted for baseline age, sex, socioeconomic status, addiction level, time since last quit attempt, outcome variable measured at baseline, total current and past two months anti-tobacco media campaign gross rating points (GRPs), plain packaging implementation, cigarette costliness, and number of days between baseline and follow-up interview.

* p-value <0.05

**Table 3:** Associations between baseline social norms, and quitting-related behaviours between baseline and follow-up.

	Since baseline, engaged in smoking limiting behaviours N (weighted) = 1, 373	Since baseline, discussed quitting with family or friends N (weighted) = 1, 373	Since baseline, sought help to quit and/or used NRT or quit smoking medication N (weighted) = 1, 373	Since baseline, attempted to quit N (weighted) = 1, 373
	Odds ratio (95% CI)	Odds ratio (95% CI)	Odds ratio (95% CI)	Odds ratio (95% CI)
Friends and family disapprove of my smoking	1.50 (1.08, 2.07)*	1.31 (0.91, 1.89)	1.26 (0.82, 1.94)	1.29 (0.67, 2.49)
Feel embarrassed to tell people I’m a smoker	2.43 (1.68, 3.49)*	1.43 (1.01, 2.02)*	1.18 (0.74, 1.88)	2.15 (1.19, 3.88)*
Quitting activity in the household	1.78 (1.00, 3.17)*	1.72 (0.92, 3.25)	1.30 (0.66, 2.57)	1.30 (0.46, 3.68)

NRT, nicotine replacement therapy

Each social norm was examined in a separate model. All models were adjusted for baseline age, sex, socioeconomic status, addiction level, time since last quit attempt, outcome variable measured at baseline (except for engaged in smoking limiting behaviours, for which baseline measures were not available), total current and past two months anti-tobacco media campaign gross rating points (GRPs), plain packaging implementation, cigarette costliness, and number of days between baseline and follow-up interview.

* p-value <0.05

**Fig 1:**
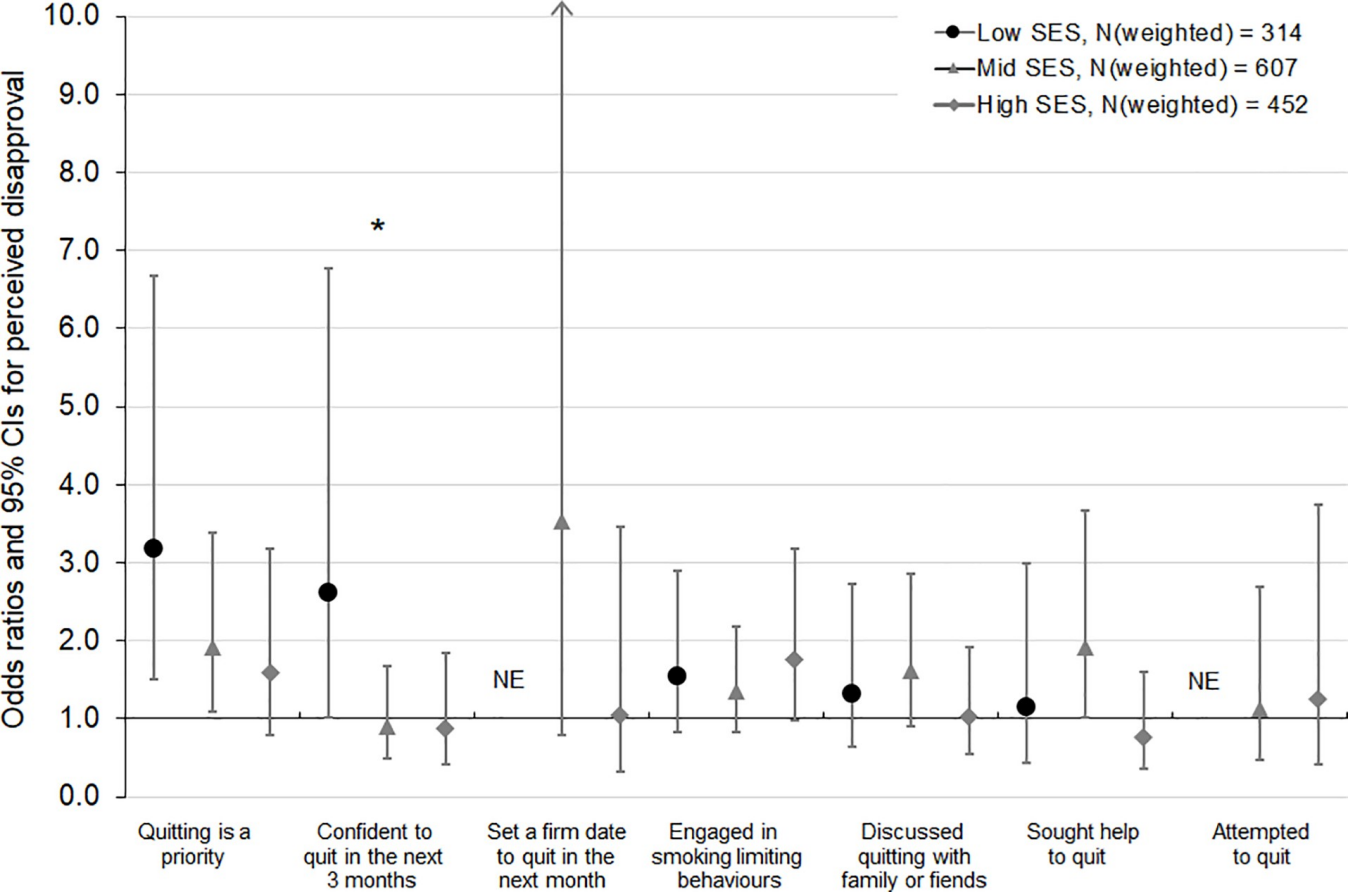
Associations of agreeing that “my closest family or friends disapprove of my smoking” (versus not agreeing to perceived disapproval) with quitting-related thoughts and behaviours, by socioeconomic status (SES). NE, not estimable due to zero cell count Each model was adjusted for baseline age, sex, addiction level, time since last quit attempt, outcome variable measured at baseline (except for engaged in smoking limiting behaviours, for which baseline measures were not available), total current and past two months anti-tobacco media campaign gross rating points (GRPs), plain packaging implementation, cigarette costliness, and number of days between baseline and follow-up interview. * p-value for interaction <0.10.

**Fig 2:**
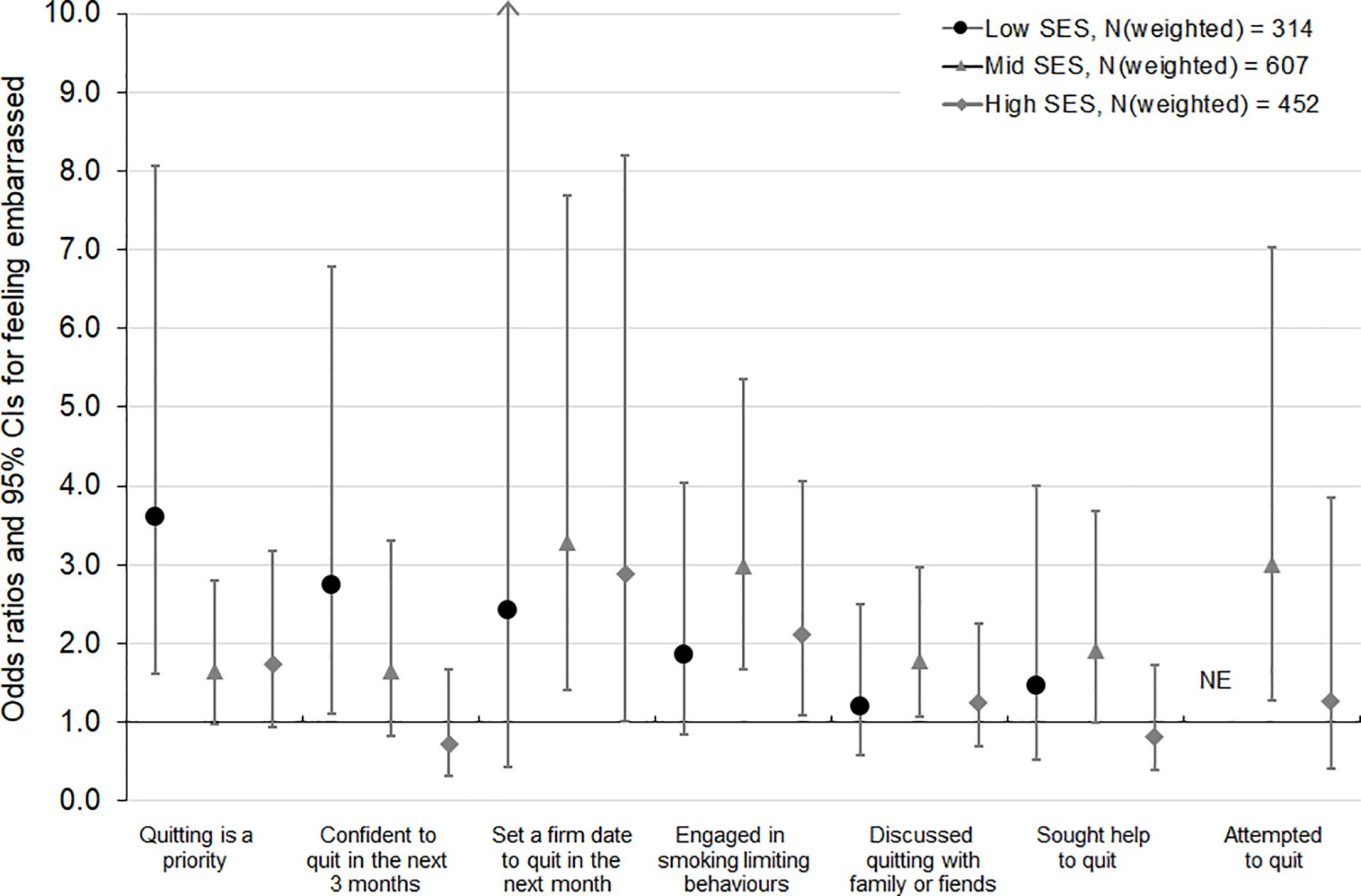
Associations of agreeing that “I feel embarrassed to tell people I'm a smoker” (versus not agreeing to feeling embarrassed) with quitting-related thoughts and behaviours, by socioeconomic status (SES). NE, not estimable due to zero cell count. Each model was adjusted for baseline age, sex, addiction level, time since last quit attempt, outcome variable measured at baseline (except for engaged in smoking limiting behaviours, for which baseline measures were not available), total current and past two months anti-tobacco media campaign gross rating points (GRPs), plain packaging implementation, cigarette costliness, and number of days between baseline and follow-up interview.

**Fig 3:**
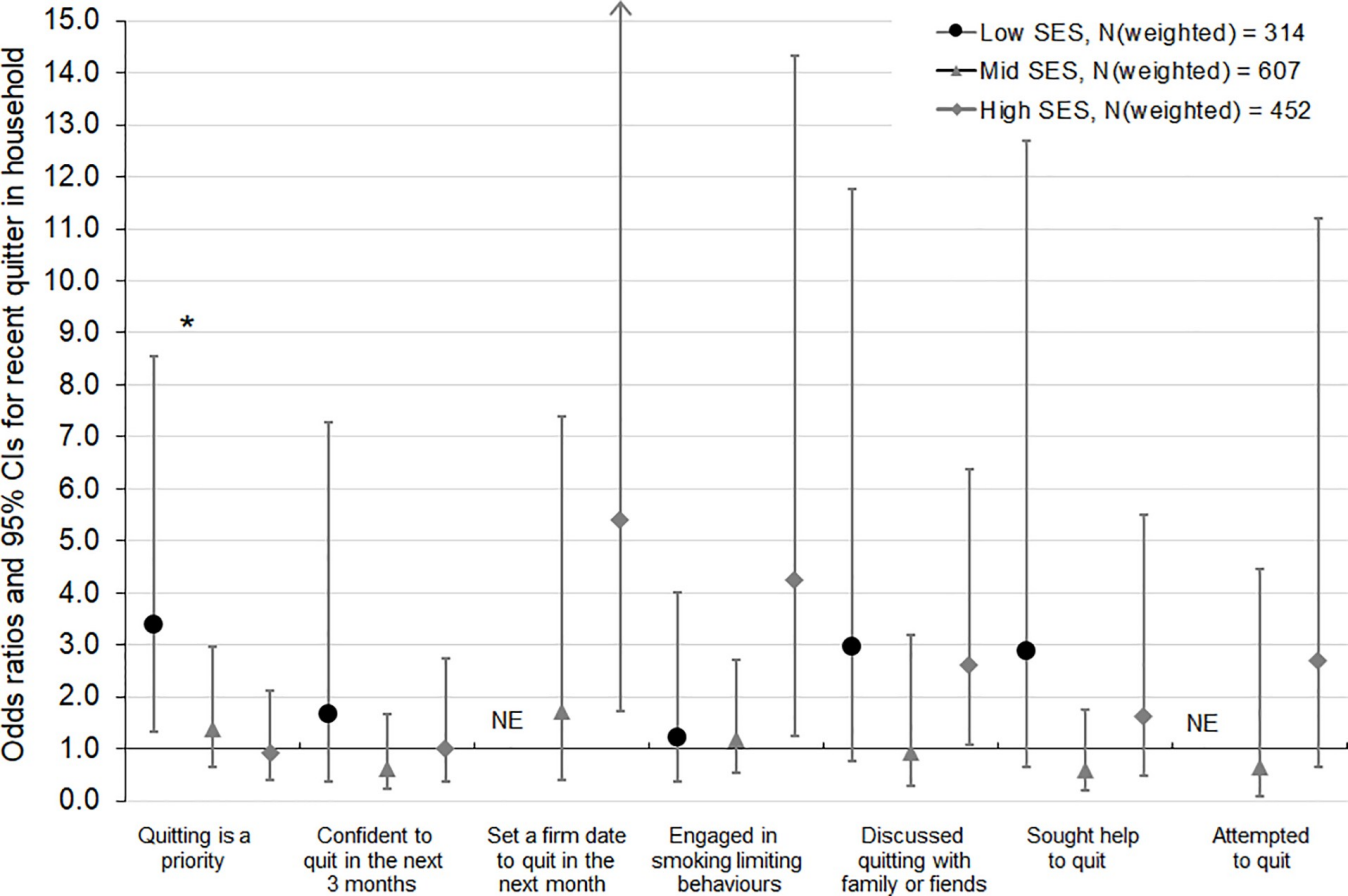
Associations of quitting activity in the household (versus no quitting activity) with quitting-related thoughts and behaviours, by socioeconomic status (SES). NE, not estimable due to zero cell count. Each model was adjusted for baseline age, sex, addiction level, time since last quit attempt, outcome variable measured at baseline (except for engaged in smoking limiting behaviours, for which baseline measures were not available), total current and past two months anti-tobacco media campaign gross rating points (GRPs), plain packaging implementation, cigarette costliness, and number of days between baseline and follow-up interview. * p-value for interaction <0.10.

Based on interaction tests, these findings did not significantly differ for low, mid and high SES smokers (p-values for interaction >0.10) (**Figs 1, 2 and 3**), with two exceptions. Interaction tests indicated a difference by SES in the relationships between disapproval and confidence to quit (Wald test F = 2.97, p = 0.05) (Fig 1) and between quitting activity in the household and quitting priority (Wald test F = 2.66, p = 0.07) (Fig 3). Stratified analyses indicated that disapproval increased confidence to quit among low SES smokers (OR 2.62 [95% CI 1.01, 6.78]), but not among mid and high SES smokers (OR 0.91 [95% CI 0.50, 1.68] and OR 0.87 [95% CI 0.41, 1.85], respectively) (Fig 1). Low SES smokers who lived with a recent quitter were more likely to have quitting as a priority (OR 3.38 [95% CI 1.34, 8.53]), whereas quitting activity in the household was less strongly and not significantly associated with quitting priority among mid and high SES smokers (OR 1.39 [95% CI 0.65, 2.96] and OR 0.93 [95% CI 0.41, 2.11]) (Fig 3). Similar patterns of larger effects on confidence to quit and on quitting priority among low compared with mid and high SES smokers were found for the other social norms, although these differences were not statistically significant based on interaction tests. Moreover, in line with these findings using an SES measure that combined information on individual-level education and on area-level disadvantage, findings using either of these SES indicators also showed that associations were mostly comparable for lower and higher SES smokers, with no significant negative effects among subgroups.

## Discussion

Findings from this population-based study of adult smokers showed that anti-smoking social norms were prospectively associated with increased quitting-related cognitions and behaviours over at least one week of follow-up. Perceiving family or friends’ disapproval, feeling embarrassed about being a smoker and living with a recent quitter each increased the likelihood of setting a firm date to quit in the next month and of engaging in smoking limiting behaviours. Embarrassment also predicted discussing quitting with family or friends and making a quit attempt between baseline and follow-up. These effects were independent of the effects of the tobacco control policy changes that occurred over this period. The associations were mostly consistent across SES subgroups, except that effects of disapproval on increasing confidence to quit, and of household quitting activity on quitting priority, were stronger among lower compared with higher SES smokers. Importantly, we did not find evidence for a negative impact of anti-tobacco social norms among low or high SES smokers on any of the quitting-related outcomes that were examined.

In line with indications from previous studies, we found that the internalisation of social norms against smoking, evidenced by smokers feeling embarrassed to tell people they are a smoker, was a stronger predictor of quitting intentions, discussions and behaviours compared with perceiving disapproval from close family and friends or living with a recent quitter [2, 15]. Although all three social norms each predicted several quitting-related outcomes, embarrassment, rather than disapproval or household quitting activity, was a stronger predictor of behavioural outcomes including engaging in smoking limiting behaviours, discussing quitting with family or friends and making a quit attempt. Smokers who feel embarrassed may not only be aware of anti-smoking norms, but they are likely to also have reflected on their own behaviour and subsequently self-generated negative emotions [2, 15], which may increase quitting motivation and behaviours. In comparison, some smokers who are aware of family and friends’ opinions about their smoking behaviour or who observe others’ quitting activity but do not relate to or do not internalise these opinions and actions, may not experience strong negative emotions about their smoking, such that they are therefore less likely to be motivated to attempt behaviour change [2, 15].

The finding that household quitting activity was associated with fewer quitting-related outcomes compared with disapproval and embarrassment is also in line with limited previous evidence which has shown that subjective and injunctive norms were more strongly associated with quitting-related cognitions and behaviours than descriptive norms [12, 40]. A cross-sectional study conducted among Dutch smokers examined the influence on quit intentions within the next three months of a range of social norms, including subjective (perceived disapproval of smoking from close others), injunctive (perceived acceptability of smoking in social situations) and descriptive norms (number of people in close social environment who smoke, and who have recently tried to quit) [12]. All social norms were associated with quit intentions, although this was most strongly the case for the subjective and injunctive norms, followed by the descriptive norms [12]. Moreover, findings from a longitudinal study of over 13,000 French smokers suggest that motivation or pressure to quit from others, but not having a smoke-free social network, was associated with abstinence after one month of follow-up [40].

Our findings also support studies that have found that higher SES smokers are more likely to perceive anti-smoking social norms compared with lower SES smokers [9, 28], although these SES differences were not statistically significant in our study. Despite suggestions from previous qualitative and cross-sectional studies, our results do not support concerns that anti-tobacco social norms may have a negative impact on smoking cessation thoughts and behaviours among lower SES smokers or enhance cessation more so among higher SES smokers. Although qualitative and cross-sectional quantitative studies suggested that self-blame and guilt about smoking behaviour may lead to avoidance of seeking help [24], findings from our study showed that there were no associations (positive or negative) between anti-smoking social norms and help-seeking behaviours, and no evidence of an undesirable effect of any of the anti-smoking social norms on cessation thoughts or behaviours among lower or higher SES smokers.

There were however differences in the magnitude of the positive effects of social norms between lower and higher SES smokers on two of the seven quitting-related cognition and behaviour outcomes. Interactions between disapproval and quit confidence, and between household quitting activity and quitting priority, indicated that associations may be stronger among low compared with mid and high SES smokers. Moreover, we found a consistent pattern of larger effects on these two outcomes among low SES smokers across all social norms. These findings therefore suggest that anti-smoking subjective and injunctive social norms may particularly help lower SES smokers to prioritise smoking cessation and to be highly confident that they could quit smoking for good in the next three months. While there were also differences in the strength of associations for other outcomes, these were not statistically significant and not consistent across social norms. Moreover, confidence intervals are wide and largely overlap across SES subgroups, and these findings should therefore be interpreted with caution.

Strengths of our study include the use of data from a broadly representative sample of smokers, and the prospective design of the study with smokers followed up around one week after the baseline interview. This allowed us to examine associations of baseline anti-smoking social norms with quitting-related self-efficacy, urgency, intentions and behaviours at follow-up, independent of similar behaviours at baseline, strengthening our confidence in the proposed causal order of effects. Our analyses were adjusted for a range of demographic characteristics, addiction level and tobacco control policies, to account for differences in these factors among smokers who participated in the study at different times between 2012 and 2014 and their effect as predictors of the outcomes.

Our study also has limitations. At baseline, less than half of people (42%) who were approached for the interview, agreed to participate. The sample was weighted to be representative of the Victorian population of smokers, thereby enhancing representativeness of the survey and generalisability of the findings. However, we acknowledge there may be unobserved factors associated with the decision to complete the survey and with quitting-related outcomes, which may have influenced our findings. A common problem with longitudinal studies is the drop-out of participants during follow-up, which could have affected our results. Participants who were lost at follow-up were more likely to be younger and to have low addiction levels, compared with smokers who were eligible for follow-up but did not participate. However, the prevalence of social norms was not significantly different among smokers who were included in this study compared with those who were lost at follow-up. Another limitation of our study is the relatively short follow-up period of approximately one week after which only a small proportion of baseline smokers were abstinent from smoking. We were therefore not able to examine the effects of disapproval, embarrassment and household quitting activity on sustained quitting success. Our findings showed effects on multiple quitting-related attitudes and behaviours including setting a firm date to quit, engaging in smoking limiting behaviours, and discussing quitting with family or friends, and making a quit attempt, increasing the confidence that our findings are not spurious. We are aware of one recent cohort study that examined stigma in relation with sustained quitting, showing that smokers in Mexico who believed that smokers are increasingly marginalised were less likely to quit successfully, while there was no association among smokers in Uruguay [41]. Further long-term follow-up studies are therefore needed to examine links between social norms and stigma and subsequent sustained quitting.

Another limitation of our analysis is that the indicators of social norms that we examined were limited to disapproval, embarrassment and living in a household with a recent quitter. Social norms and tobacco denormalisation are broad concepts, and future studies should therefore measure and examine the relative effects of various other aspects of social influences including other subjective social norms (such as individual’s perceptions of what friends, parents, partners, children and the broader societal community think they should or should not do), other indicators of internalised social norms (including negative emotions such as guilt and shame about smoking), which have been suggested to have differential effects on behaviour [15, 42–44]. Moreover, the influence of ingroup norms (norms in a social group to which a person psychologically identifies as a member) and social identity in driving behaviour change could be further examined [2].

Lastly, it should also be noted that our results may not be generalisable to smokers from other countries. The levels of disapproval and embarrassment in our study population have likely been influenced by the strong tobacco control policies implemented in Australia over the past decades [45]. Similar studies are therefore needed among socioeconomically diverse smokers in other countries where there are fewer restrictions on public smoking and higher smoking prevalence, to examine if they find consistent effects of social disapproval of smoking and embarrassment to be a smoker on quitting outcomes. Future studies should also examine the extent to which policies, such as advertising, display and packaging laws (e.g., pictorial health warnings on packs and plain packaging) which aim to limit the influence of tobacco industry marketing and branding, anti-smoking mass media campaigns and cigarette cost increases, have helped to change social norms about smoking.

In summary, our findings among Victorian adult smokers show that perceiving disapproval of smoking behaviour from family and friends, feeling embarrassed to be a smoker and living with a recent quitter are linked with positive quitting-related cognitions and behaviours. Our findings therefore suggest that if existing and new tobacco control policies increase levels of anti-smoking social norms they may contribute to an environment in which smoking is less socially supported and in turn more supportive of quitting [1]. Contrary to suggestions that social norms may have negative effects on lower SES smokers’ motivation to quit, the effect of social norms tended to be stronger among low SES smokers in terms of increasing quitting confidence and quitting as a priority. This suggests there should not be reason for concern about the potential for negative effects on cessation behaviours among smokers across all SES levels. Instead, increasing levels of subjective and injunctive social norms may particularly help lower SES smokers to try to quit.
